# Assessment of Community Stakeholders’ and Health Educators and Professionals’ Needs for the Continuous Enhancement of Sexual and Reproductive Health and Rights in Mali (Project CLEFS): Protocol for a Convergent Mixed Methods Study

**DOI:** 10.2196/64796

**Published:** 2025-04-01

**Authors:** Sabina Abou Malham, Doufain Traoré, Fatoumata Dicko, Gabriel Blouin Genest, Jennyfer Boudreau, Drissa Mansa Sidibé, Souleymane Sidibé, Issa Souleymane Goïta, Aminata Sangaré, Mohamed Togo, Delphine Diarra, Michèle Rietmann, Mahamane Mahamoudou Maïga, Suzie Boulanger, Ann Isabelle Grégoire, David-Martin Milot, Djamal Berbiche, Sarah Stecko

**Affiliations:** 1 Centre interdisciplinaire de développement international en santé (CIDIS), School of Nursing Faculté de Médecine et des Sciences de la Santé, Université de Sherbrooke Longueuil, QC Canada; 2 Santé Monde Bamako Mali; 3 Faculté de Médecine et d'Odonto-Stomatologie Université des Sciences, des Techniques et des Technologies de Bamako (USTTB) Bamako Mali; 4 Centre interdisciplinaire de développement international en santé (CIDIS) Faculté de Médecine et des Sciences de la Santé Université de Sherbrooke Longueuil, QC Canada; 5 Institut National de Formation en Sciences de la Santé (INFSS) Bamako Mali; 6 Cégep de Saint-Jérôme Saint-Jérôme, QC Canada; 7 Centre de recherche Charles Lemoyne Faculté de Médecine et des Sciences de la Santé Université de Sherbrooke Longueuil, QC Canada

**Keywords:** mixed methods study, protocol, sexual and reproductive health and rights, Mali, primary health care providers, women and girls’ health needs

## Abstract

**Background:**

In Mali, a lack of qualified human resources in primary health care and sexual and reproductive health and rights (SRHR) is one of the greatest barriers to the population’s access to standard health services. Frontline professional training must be strengthened to respond to the needs of the population, particularly those of women and girls. Training must be conducted using an interdisciplinary and adapted approach to promote gender equality.

**Objective:**

This study aims to identify the SRHR training needs among the community, educational actors, and primary health care providers.

**Methods:**

A concurrent mixed methods design was adopted, using 2 methods. A quantitative method, through a cross-sectional analytical survey, will be conducted at the community level with university community health centers (CSCom-U) users and adolescents in CSCom-U health areas, as well as at the health education institution and community health centers levels with teachers, students, and interdisciplinary professional groups within the CSCom-U and district hospital maternity. Descriptive and inferential analyses will be conducted to process quantitative data. This research is at the stage of data analysis and interpretation. A qualitative method, based on 3 sources of data (focus groups, individual semistructured interviews, and document analysis), which involved the same targets as the quantitative component, with additional community actors such as Community Health Associations (Associations de santé communautaire) and Women’s Service User Communities. A thematic analysis of the qualitative data using a mixed deductive and inductive method will be performed.

**Results:**

*:* Field data collection took place from March to April 2022. Quantitative data from 3153 participants are being analyzed using SPSS. Qualitative data from 11 interviews and 27 focus groups were processed with Qualitative Data Analysis Miner. Data analysis is still ongoing.

**Conclusions:**

This study will provide a better understanding of adolescents and SRHR user’s service needs in terms of health services availability and quality and SRHR knowledge, issues related to student training quality, the level of adequacy between the training offered and the actual needs of the service recipients, and the level of preparation and ability of teachers to provide quality teaching taking gender equity into account. The recommendations drawn from this assessment will propose concrete actions to improve women and girls’ health services provided by professionals, and to better adapt the future health professionals’ profiles to the needs of communities, particularly those of women and girls.

**International Registered Report Identifier (IRRID):**

DERR1-10.2196/64796

## Introduction

In Mali, the health system does not yet have sufficient capacity to meet the health needs of the population, especially those of women and girls. Primary health care (PHC), considered to be the foundation of sexual and reproductive health and rights (SRHR) service delivery, remains poor [1]. Each year, 33% of pregnant women give birth outside of a health facility, and the maternal death ratio is 440 out of 100,000 births [2]. Although 24% of Malian women aged between 15 and 49 years report having unmet family planning (FP) needs, only 15% of them use modern contraceptive methods [3]. Among women with unmet FP needs, 17% are oriented toward pregnancy spacing [3].

In rural areas, where approximately 75% of the Malian population live, access to PHC services is more difficult, thus creating substantial inequities [4]. There is a significantly higher fertility rate (6.8 vs 4.9 in urban areas), a lower proportion of prenatal care visits (37% vs 67% in urban areas), a higher proportion of deliveries outside a health facility (39% vs 7% in urban areas), as well as more limited access to FP (contraceptive prevalence rate for sexually active women not in a union is of 27% vs 44% in urban areas) [3]. This highlights that women and girls have poor access to nonstigmatizing SRHR services and information as well as insufficient bargaining power regarding their sexual and reproductive health [5,6]. In addition, gender-based violence (GBV), including domestic violence, is also widely common as 45% of women aged 15-49 years are survivors of physical or sexual violence [3]. However, this violence remains poorly documented due to several barriers such as low institutional commitment to integrating this issue, particularly in health reporting. There is also a lack of health professionals training and clinical screening tools for mental health problems, particularly for postpartum depression, GBV survivors, and people affected by armed conflict [7].

Health education institutions in Mali lack the human, material, and financial resources to fulfill their role in teaching PHC and SRHR care [8]. The gaps in undergraduate and continuing education contribute to the lack of skilled human resources in PHC and SRHR, particularly in rural areas [9]. Skills of frontline professionals need to be strengthened at the level of both institutional management and training, to respond to the population’s needs using an interdisciplinary approach, to promote gender equality (GE), and, ultimately, to offer training tailored to the needs of the labor market.

To address these concerns, the Canadian consortium—which is composed of Santé Monde, the Cégep de Saint Jérôme and the Centre interdisciplinaire de développement international en santé from University of Sherbrooke and funded by Global Affairs Canada—has implemented the Local Teaching Communities for Healthy Women and Girls (Communautés locales d’enseignement pour les femmes et les filles en santé [CLEFS; French]) project. This project will operate for a 5-year period (2020-2025) in 5 different regions of Mali (Kayes, Koulikoro, Ségou, Sikasso, and the district of Bamako). The CLEFS project was developed in partnership with the Malian Ministries of Health, Higher Education, and Women’s Promotion and with health training institutions in Mali (Faculty of Medicine and Odontostomatology [FMOS], National Institute for Training in Health Sciences [Institut National de Formation en Science de la Santé [INFSS]], and private schools), women’s user committees, persons in charge at Community health associations (Associations de santé communautaire [ASACO]) for university community health centers (CSCom-U), as well as the Malian district hospitals to which the CSCom-U are attached. CSCom-U is a first-level health center in community settings offering a minimum package of health care. As community health centers with a university orientation, they also offer delocalized internships for medical students.

This project is based on the principle that health education institutions in Mali must fully integrate PHC and SRHR concepts into their curricula and pedagogical approaches to fulfill their social responsibility toward women’s and girls’ needs. This process involves collecting and analyzing data on the unmet needs of the beneficiaries, as well as those related to training and frontline health care system actors. In this context, the CLEFS project supported the Malian health training institutions (INFSS and FMOS) in conducting a participatory evaluation.

## Methods

### Research for the Continuous Improvement of PHC and SRH Training Programs

Improving care in SRHR requires going through all the steps of the training engineering process to ensure continuous improvement of health care services and related training. A key step is conducting a participatory evaluative study to identify the SRHR needs of the communities served by the CSCom-Us, with a focus on women and girls, as well as those of the various actors involved in training and in the provision of PHC services [10,11] to assess the adequacy between the real needs of the communities and the targeted output profiles in SRHR.

SRHR training needs for front-line care providers are those focusing on essential services such as comprehensive sexual health education, FP, antenatal, intrapartum, and postnatal care including emergency obstetric and newborn care, prevention and treatment of HIV and other sexually transmitted infections (STIs), postabortion care, and prevention, immediate services and referrals for cases of GBV, and finally prevention, detection, and management of reproductive cancers [12]. Training needs encompass a patient-centered approach that respects individual gender considerations and human rights.

This participatory approach will allow a better involvement and ownership of the needs assessment methodology by Malian professionals, as well as a better acceptance and use of the resulting findings. The results will reflect a concerted and consensual identification of SRHR needs and therefore an appropriate definition of the expected output profiles at the level of both undergraduate and continuing education.

### Study Objectives

#### Overview

The main objective of this study is to depict the real and differentiated needs of Malian actors and primary care users related to SRHR to improve the training programs and services offered in this area.

This translated into the following specific objectives as follows: (1) to identify the SRHR needs of community actors and beneficiaries (adolescents and CSCom-U users); (2) to identify the SRHR training needs of students, trainers, and health professionals involved in training in terms of SRHR capacity building; and (3) to improve the curricula of nursing and obstetric practitioners in accordance with the needs of the health care professionals and specific needs of the populations served, in particular those of women and girls.

#### Overall Study Design

Using a participatory approach, this needs assessment adopts a concurrent mixed methods research design combining quantitative and qualitative methods [13]. It is based on the convergence of the analytical results performed for each method with all the actors concerned [14,15].

### Method 1: Quantitative

#### Quantitative Study Design

Cross-sectional surveys were conducted in the 5 project intervention zones, namely the cercles of Sikasso, Kolokani, Baraouéli, Kayes, and Bamako in the regions of Sikasso, Koulikoro, Segou, Kayes, and Bamako. Note that a cercle is the second administrative division of Mali. Mali is composed of 8 regions and 1 capital district (Bamako), with each region divided into 49 cercles.

Three levels were considered as follows: (1) community level: targeting CSCom-U users and adolescents in the CSCom-U health areas; (2) health education institutions level (FMOS and the main INFSS and its regional branch schools [called Annex]) and CSCom-U targeting teachers, students/trainees as well as; and (3) groups of interdisciplinary professionals from CSCom-U and the maternity ward of district hospitals supported by the CLEFS project.

#### Participant Inclusion Criteria

The study participant inclusion criteria are as follows.

1. Female users: women of childbearing age between 15 and 49 years old who have resided in the household during the last 6 months before the survey.
2. Male users: male heads of household aged 20 years and older or their legal representative aged 20 years and older who have stayed in the household during the last 6 months.
3. Adolescents: unmarried girls and boys aged 15-19 years.
4. Interdisciplinary professional groups: consist of physicians, clinical supervisors, midwives, and obstetric nurses practicing in CSCom-U and district hospitals maternity units for at least 6 months prior to the survey.
5. Teachers: lecturers and supervisors on a temporary or contractual basis in the nursing care and obstetrical care programs at the INFSS or Annex schools and in the family/community medicine programs at the FMOS, who have been teaching for at least 1 year before the survey.
6. Students: students in the nursing care/obstetrical care streams of the INFSS and its Annex schools, as well as those of the Specialized Studies Diploma in Family/Community Medicine programs, from the 2nd year of teaching onwards—including trainees—with a proportional distribution between men and women and between the different years of study.

#### Sample Size and Techniques

Sample sizes of the different community targets and intervention zone were calculated separately based on the total number of households in the health areas and the size of the population targeted by the survey. Sample size was, considering the proportion of the phenomenon studied, at 50% and the CI at 95% [16]. The choice of 50% value allows us to have a sufficiently large sample to make inferences, regardless of the true value of the phenomenon studied in the real population. It was calculated using Epi Info7 software (Centers for Disease Control and Prevention) and was distributed proportionally to the size of the households in the selected villages.

Student and teacher sample size was calculated separately based on the total number of students in the nurse and obstetrical care stream at INFSS and its Annex schools and the number of family/community medicine and teachers involved in these streams at the 2 institutions. The calculation was done using the same method and the same software as the community-level actors in a separate way for the INFSS, the INFSS Annexes, and the FMOS. Thus, the calculated sample size (teachers and students) at the Annex schools’ level was proportionally distributed based on the number of teachers and students in the different Annexes.

#### At the Community Level

A multistage sampling technique was used for selecting study participants (stratified random, clusters, and simple random).

Stratified random sampling was performed in the villages or sectors of the CSCom-U health catchment areas considered as natural strata (4 per CSCom-U). The lists of villages in the health catchment areas were established by CSCom-U. Strata were randomly drawn considering proportionality in this population by applying the systematic random method with a sampling step calculation. Once these strata were randomly drawn, the households within these villages or sectors were grouped into clusters (composed of a maximum of 40 households) using the segmentation method for each one. Thus, clusters were randomly selected, and the survey was conducted among the households in these clusters.

Another simple random draw was then conducted within households to select one woman of reproductive age in each household (if there are several) to answer the user survey questions. The head of the household or his/her representative who is 20 years or older was surveyed to answer questions about users. Adolescents (male/female [M/F]) meeting the criteria in the selected households, were also surveyed after a random draw if numerous. If the number of households expected to be surveyed was greater than the number of households in the selected cluster, another cluster was randomly selected to complete the total number of samples required for the survey.

#### At the Educational Institutions and CSCom-U Levels

A simple random draw without replacement was made from a list of all teachers of the Specialized Diploma of Family Medicine/Community Medicine and the INFSS and Annex schools to obtain the required sample size. Another simple random draw without replacement was also carried out based on the list of the students of the cycles and the level of teaching concerned to obtain the required sample size. The sample size was segregated by gender/level/profession. Randomly selected students were contacted to participate in the survey after obtaining their consent. If a student refused to participate in the survey, another number was randomly drawn to replace him/her until the total sample size was reached.

For the interdisciplinary focus groups in the CSCom-U and district hospitals, as the number of staff is not large, all physicians, midwives, and nurses with a role in SRHR who were available at the time of the team’s visit and who gave their consent to participate were surveyed.

There is a low number of professionals in CSCom-U and maternity care. Therefore, all available and willing physicians, midwives, and nurses were solicited for the CSCom-U and district hospitals interdisciplinary professional focus groups.

#### Quantitative Data Collection and Management

To carry out the data collection, teams of external interviewers and trained supervisors were deployed on site. The interviewers were external individuals with expertise in conducting surveys and were recruited by the project team for the period of the data collection. They were responsible for carrying out the surveys among users, adolescents, and students. Due to the sensitivity of sexual and reproductive health issues, female interviewers conducted the surveys among female respondents and male interviewers among male ones.

Supervisors made up of members of the State’s Technical Services and members of the FMOS, INFSS, and Association of Private Health Schools were responsible for verifying the conformity of investigators’ work and supporting them when necessary. Their involvement strengthened the process of ownership of the continuous quality improvement approach from data collection to the presentation of evaluation results. This approach ensured the transfer of acquired skills to other stakeholders and replicated the continuous quality improvement process in their respective regions, with little or no outside technical assistance. Supervisors were also responsible for conducting the qualitative component.

The FMOS experts (Mali), in collaboration with the INFSS (Mali) and supported by the CLEFS project team (Canada), developed and customized questionnaires tailored to the various survey targets. These questionnaires were subsequently distributed to all stakeholders involved in the training needs assessment process for review, appropriation, and validation of the tools. Once validated, the tools were created on the Kobocollect platform (KoboToolbox software) and then deployed on tablets or smartphones to collect data. This method offers several advantages such as quickness, error avoidance, the obligation to answer all questions (better completeness of data), and reduction of outliers. All data collected on-site were sent daily to the Kobocollect account and extracted in an Excel file to be reviewed during the data collection.

Prior to data collection, a 3-day training was conducted for the entire evaluation team. This was followed by a 1-day pretest in an area outside of the project’s intervention regions to assess its clarity and comprehensibility and to enable the methodology to be adjusted if needed.

#### Quantitative Data Analysis

Since the data were collected with tablets or smartphones, data entry was instantaneous. Data were extracted from the KoboCollect platform in Excel format, then processed, cleaned, and finally analyzed using Excel (Microsoft Corp) and SPSS software (version 27; IBM Corp).

Data analysis and interpretation are still ongoing. According to our research protocol, we will conduct descriptive analyses, such as examining proportions, averages, and SDs, to gain insights into sociodemographic characteristics. This includes examining levels of knowledge, attitudes, practices, and perceptions regarding various aspects of SRHR, specifically FP, sexuality, STIs, HIV, prenatal care, childbirth, and GBV. The level of satisfaction and challenges related to the use of CSCom-U will also be analyzed. Access to information for CSCom-U users and adolescents regarding SRHR will also be measured.

The evaluation will also include an assessment of the satisfaction levels of students with the SRHR programs attended, the training environment and internships including safety aspects; students’ sense of preparedness to face professional life; level of preparedness of teachers and health professionals to respond to the needs of students and interns and to train students able to respond to community needs.

Several inferential statistics will be used. For example, chi-square tests will be used to compare the proportions of the variables of interest and the student *t* test will be used to compare mean scores. Gender analyses will also be performed to understand gender specificities. The statistical significance level will be set at 5%. Multivariable regression analysis will be conducted afterwards if significant associations are found in bivariate analyses.

### Method 2: Qualitative

#### Qualitative Study Design

Three groups (ie, multidisciplinary teams, community stakeholders, teaching teams, and students) were targeted for the qualitative component, which was conducted using 3 sources of data: focus groups, semistructured individual interviews, and a literature review.

#### Participant Selection and Method

##### Community Actors

Community actors mainly included the project’s implementation partners, that is, ASACO members, women’s user communities, youth groups, and community relays. This phase also reached users, adolescents, traditional birth attendants, traditional therapists, and community leaders. Targets were chosen according to a purposive sampling and snowball strategy until data saturation was reached according to the inclusion criteria previously identified in the sample of the quantitative component. To ensure a better representation of this population, we aim to select a variety of profiles in terms of age, gender, number of years in office, and as members of different associations.

At the community level, 6 focus groups were conducted as well as semistructured interviews per health area as follows: a mixed focus group (M/F) with members of each ASACO (8 to 12 people); a women’s focus group with communities of women users (8 to 12 people); a mixed (M/F) focus group with youth groups, provided that heterogeneous youth groups exist in the localities. If not, or depending on cultural realities, homogeneous groups were conducted; a mixed focus group (M/F) with community relays of 8 to 12 people; a homogeneous focus group with women users of the SRHR services of the CSCom-U; a mixed focus group (M/F) with adolescents; and individual interviews with a traditional birth attendant or rural maternity officer and a traditional therapist per village/area, if available.

##### CScom-U and District Hospital Maternity Units’ Multidisciplinary Teams

These multidisciplinary teams were composed of medical doctors, midwives, and obstetric nurses involved in the SRHR services. CSCom-U and maternity technical staff were not numerous and therefore all of them were involved.

Concerning the data, a mixed (M/F) focus group was organized at each CSCom-U and district hospital maternity unit to explore the following elements: the clinical skills of health professionals, the challenges, and issues they face in providing quality SRHR services to women and girls, their level of ability (training, tools, and equipment) to provide quality SRHR services as well as mental and environmental health issues. Discussions also focused on their ability to provide support and respond to the needs of trainees, including safety issues, and harassment.

##### Educational Institutions Actors

#### Overview

Two groups of actors were targeted at this level: teachers and students. They were chosen according to a purposive sample strategy and until data saturation was reached, according to the inclusion criteria already identified in the sample of the quantitative component. To ensure a better representativeness of this population, we selected a variety of profiles in terms of gender, seniority, level of study, teaching field, and type of teacher (lecturer and supervisors).

#### For the Teachers

Three mixed (M/F) focus groups (1/nursing, 1/obstetrical, and 1 family or community medicine specialty diploma) of 8 to 12 teachers were organized. Our objective was to better understand the level of preparation of the teachers in terms of pedagogy, teaching SRHR, their expectations, and the difficulties they face in teaching in the continuous improvement of programs.

#### For Students

A heterogeneous focus group for the nursing stream, a homogeneous focus group for the obstetrical stream, and a heterogeneous focus group for the family or community medicine specialty diploma of 8-12 students was organized. Our objective was the following: to identify the main challenges and issues related to the quality of training, the adequacy between training and the needs of the populations, the training environment, GBV, security aspects, and the consideration of their specific needs by teachers. To obtain quality information, students were selected from the 2nd year of training.

##### Document Analysis

In addition to the literature review, a documentary analysis was conducted and covered: the CSCom-U supervision reports, maternal death audits, CSCom-U and district hospitals activity reports, previous evaluation reports, etc. These reports helped to determine the needs already identified by the national or regional level at the CSCom-U and to understand the underlying problems related to SRHR.

#### Qualitative Data Collection and Management

##### Overview

Focus groups and individual interviews were conducted using interview guides tailored for each target group. They were developed by the project team in collaboration with key partners from FMOS and INFSS and validated by all stakeholders in the needs assessment process. The guides were inspired and adapted to the context and needs of the project based on existing guides such as the Demographic and Health Survey and covered the main themes explored (eg, SRHR knowledge and services offered) according to the actors of each target. The various individual interviews and focus groups were conducted by the survey supervisors, who are the project’s supporting partners, that is, agents of the government’s technical services, but also key partners. For the focus groups, a minimum of 2 people conducted them. One was responsible for facilitating the discussion and the other for managing the recording, taking notes, and making observations of the participants. For individual interviews, 1 or 2 people conducted them depending on the number of supervisors, one of them was responsible for asking the questions, and the other for taking notes and managing the recording.

##### At the Community Level

For the recruitment and data collection, our evaluation team informed ASACOs leaders as well as the Technical Directors of the Center of the evaluation process, the date, and the participants to be met. They then informed the other members of the ASACOs, the communities of women users, the youth groups, and the community relays and set up a schedule of visits and meetings with the different participants. The different discussion groups were conducted in the ASACO offices. ASACOs, with the support of the evaluation team, made the necessary arrangements to ensure the involvement of the participants and to respect data confidentiality.

Guidebooks were written in French, but were administered in Bambara, the most widely spoken national language of Mali. The observer took notes directly in French. Each focus group lasted a maximum of 120 minutes and was recorded after obtaining verbal or written consent from the participants. Discussions were fully transcribed as they occur in verbatim form and translated by interpreters when needed.

Individual interviews were conducted with traditherapists, traditional birth attendants, and community leaders and occurred in the villages or areas selected for the quantitative survey. Interviews were held after the ASACO leaders had informed the customary authorities and obtained the free and informed consent of the participants. Interviews lasted 45 to 60 minutes at most. The interviews were conducted in Bambara and verbatims were translated into French.

##### At CScom-U and District Hospital Maternity Units’ Levels

The evaluation team informed the technical directors of the center and the district medical officer, who in turn informed the technical staff of the objectives of the evaluation, the people to be involved in the process, and the schedule for the evaluation team’s visit. In collaboration with their ASACO, they made the necessary arrangements to encourage the participation of the people concerned as well as confidentiality during the data collection.

##### At the Health Education Institution Level

Under the guidance of the evaluation team, the administration of these institutions informed faculty and students of the evaluation process, objectives, and schedule. Together they organized the recruitment of faculty and students who wished to participate in the interviews. They provided a room for the team to ensure the confidentiality of the interviews. At the school level, interviews were conducted directly in French and notes were taken in French. They were also recorded after obtaining participants’ consent.

#### Qualitative Data Analysis Methods

Data analysis and interpretation are still ongoing. Qualitative data analysis will be conducted using an iterative process and will include the listening of audio recordings, successive readings of French transcriptions, team coding, team analysis, and participant validation. The project team will code the interviews using QDA (Qualitative Data Analysis; Provalis research) Miner software using a mixed deductive and inductive approach. This iterative method simultaneously makes sense of the data collected based on the state of knowledge, while potentially identifying new meanings. A brief list of initial codes based on the interview guides will serve as a coding grid a priori. It will be modified and enriched as the analysis proceeds. Coding will be controlled by a double coding technique performed by a member of the project team and the expert researchers. Parallel and independent coding will be done for the first few 4 interviews, followed by a comparison of the results. This process will be repeated until a consensus list of initial codes is obtained as well as intercoder fidelity greater than 90% [17].

#### Mixed Methods Integration

**Figure 1:**
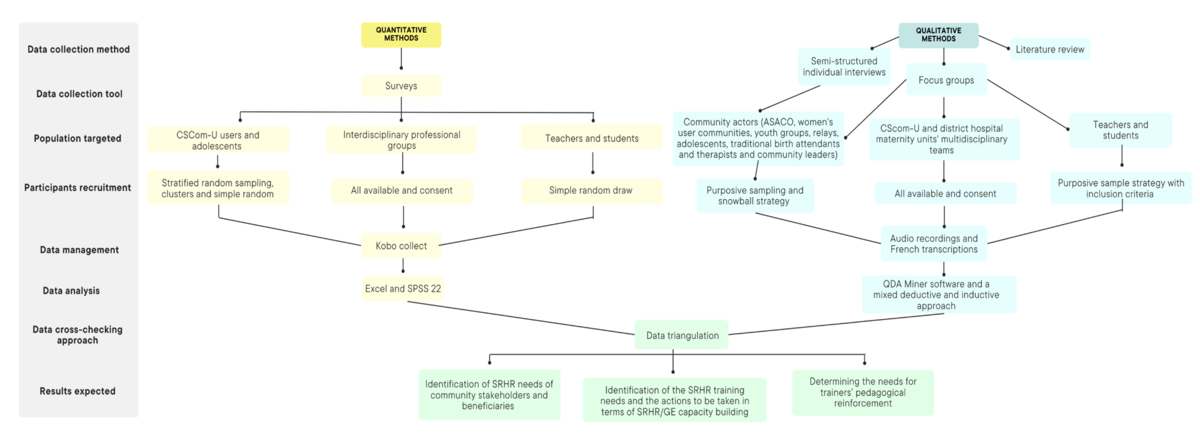
Overview of the main stages of the research study. ASACO: Associations de santé communautaire; CSCom-U: university community health centers; SRHR: sexual and reproductive health and rights.

We will adopt a strategy using the matrix technique to summarize and present the QUAN and QUAL results [18]. First, the main statistical results (QUANT component) of the surveys will be presented by the participant group. Second, the themes emerging from the QUALI component (interviews and group discussions) will also be presented in a matrix by the participant group. This will enable us to look for similarities and divergences within and across participant groups, as well as patterns between different participant groups in each component of the study. Third, our integration strategy will focus on concordance as well as divergence between the main qualitative and quantitative findings [18,19]. Thus, we will develop a mixed methods matrix combining the main QUAN and QUAL results, organizing the data according to key themes and corresponding variables to enable comprehensive analysis and interpretation. This matrix will contain a row for each key qualitative theme (ie, needs related to GBV, FP, pregnancy and neonatal care, STI prevention and treatment, postabortion care, sexual health education, clinical support for GBV survivors, and SRHR capacity building needs for trainers), which will be compared or contrasted with the corresponding quantitative results for each theme (eg, knowledge and practice related to FP, STI prevention, and needs for trainers’ pedagogical reinforcement; Figure 1).

This approach is consistent with guidance on methods that advocate complementing quantitative analyses with in-depth data obtained from focus groups and individual interviews [20,21].

#### Evaluation Team Training and Formative Research

##### Overview

Prior to the data collection start-up, a 5-day training session on the use of the QDA Miner qualitative analysis software was organized for the evaluation team in Mali to strengthen their capacity to analyze qualitative data. This training was facilitated by a consultant supported by the University of Sherbrooke who was also in charge of making the software available.

A further 3 days’ training on the KoboCollect software was provided to the evaluation team by the project’s monitoring and evaluation technical advisor as required.

Prior to the teams’ field deployment, a 4-day training session was held for all individuals involved in the needs assessment process. The training was provided by FMOS, INFSS, and the CLEFS project team and focused on the following.

##### Understanding the Roles and Responsibilities of Team Members

Following are the roles and responsibilities of the team members:

- The interviewer’s responsibilities in terms of listening, paying attention, etc.
- The pitfalls to be avoided (nonjudgmental and noninterrupting)
- Setting up conditions to ensure data confidentiality
- Seeking free and informed consent to participation
- Respecting societal and cultural values.

##### Understanding and Translating the Different Tools to Be Used

The following will be ensured in understanding and translating the tools to be used:

- A detailed explanation of the tools to be administered
- An exchange to validate the translation of these tools into the national language so that any questions asked are understood and worded correctly.

##### Survey Methodology, Including On-Site Data Collection

The survey methodology involves the following:

- The process of identifying households and survey targets
- The process of obtaining participant consent and discussing the ethical aspects of the research process
- Data collection procedures, including digital data collection (eg, downloading, filling in and saving forms, and sending final versions).

##### Managing Sensitive Cases and Difficult Situations

Sensitive cases and difficult situations will be managed as follows:

- Training on how the evaluation team should act if respondents confide to us or seek our advice or support
- Following this 3-day theoretical training, a pretest was organized on the fourth day in a Bamako commune with a dozen people to identify and rectify any errors of understanding and phrasing before the team’s deployment in the field.

#### Ethical Considerations

This study adheres to the ethical guidelines and principles relevant to human research. The ethical approval was granted by the Research Ethics Committees of the Malian FMOS (reference number: 20231 132 ICE/USTTB) and the University of Sherbrooke (reference number 2022-3261).

Prior to the teams’ deployment, the ASACO’s offices informed the local authorities in all villages or neighborhoods of the survey teams’ presence. Once on site, the teams visited these authorities to obtain their approval to conduct survey activities in their villages or neighborhoods.

Informed consent to participate from all respondents was obtained at the beginning of the survey and all individuals were fully informed of the study's purpose, potential risks, and their right to refuse to participate, to withdraw at any time, or to not answer certain questions without any justification or prejudice. If a participant wished to withdraw during or after the research, he or she had to contact the research team responsible whose contact details were provided on the consent form.

Confidentiality and anonymity of data were explained to the respondents. For minors, consent was also requested from parents or guardians. An informed consent form was developed outlining the objectives of the research, the voluntary and nonprofit nature of participation, data confidentiality and anonymity, and the approximate duration of participation. This form was shared by the collectors and its reading (in Bambara or French, as preferred) was mandatory and they were asked to devote time to explain it to the participants. The supervisors ensured that interviewers complied strictly with these instructions. All data are confidential and will be only used to achieve the project’s results.

Identifiable information will not be disclosed in written publications and oral or poster presentations.

## Results

Field data collection took place from March to April 2022. Quantitative data were collected using the KoboCollect application, and descriptive and bivariate analyses were carried out using SPSS software on a sample of 3153 participants. For qualitative data, 11 individual interviews and 27 focus groups were conducted and analyzed using QDA Miner software. Data analysis and interpretation are currently being finalized, and the first results should be submitted for publication in 2025.

## Discussion

### Overview

This research is one of the first to provide a holistic assessment and understanding of SRHR care needs across a continuum of services (from training, and provision to receiving care) covering the perspectives of various stakeholders at different levels (community, educational, and interdisciplinary health care professionals). It is also one of the first SRHR studies to be deployed in 5 regions (Bamako, Kayes, Koulikoro, Ségou, and Sikasso) across Mali.

### Expected Findings and Outcomes

The results of the needs assessment will help us to better understand the needs of adolescents and users of SRHR services, not only in terms of availability, quality of services, and responsiveness of health care providers to their specific needs, but also in terms of SRHR information and knowledge.

Results will also help us gain a better understanding of the issues related to the quality of student training and supervision, and the adequacy between the training offered and the health care needs expressed by service users. They will also enable us to assess the readiness and skills of educators to provide quality SRHR teaching while considering GE.

Studies have demonstrated the effectiveness of training interventions in improving the attitudes and practices of health professionals regarding SRHR in low- and middle-income countries [11,22]. Therefore, recommendations resulting from this needs assessment will enable us to suggest concrete interventions for health care professionals and educators, allowing them to meet the community’s needs—particularly those of women and girls.

Based on the results, we will design and implement a multifaceted strategy targeting the 3 levels (community, education, and health care providers).

In terms of training programs, the INFSS and FMOS will revise their curricula in line with the prioritized SRHR needs to fill the gaps in the existing modules. The plan is to integrate these needs into existing modules such as those concerning adolescent sexual and reproductive health, as well as care for GBV survivors and postabortion care, and to adopt interactive pedagogical methods to better prepare the future workforce in SRHR. In sum, the content of the existing curriculum modules will be repackaged to incorporate the priority needs and suggestions made by Malian participants and partners at the national, educational, and community levels.

Regarding the training needs, a package of interventions (training workshops and problem-solving exercises such as training on the management of GBV) will be developed, drawing directly on the results and recommendations of the study to enhance the teachers’ and health care professionals’ knowledge, skills and attitudes in providing SRHR services.

For community members, numerous interventions (eg, image kits, conferences, and training with peer educators on specific topics) will be codeveloped, prioritizing community involvement and ensuring that content will be oriented according to community acceptability and reflective of their needs.

Moreover, to bridge the gap between the study results and tangible impact in the field, the research findings will be disseminated through a variety of strategies and with numerous CLEFS project partners such as the relevant government actors, ASACO, community health centers, health professionals, and other community actors. This will enable key stakeholders to engage with the insights and facilitate the integration of the recommendations within their daily activities (practice and teaching).

This multifaceted strategy aims to ensure that the study results are effectively tailored to the Malian context and that they reach a wide range of stakeholders involved in PHC and in the provision of SRHR services. Having access to and using quality SRHR services are well linked to health care professionals’ competencies, GE, and moreover to the well-being, community stability, and development [11,23].

### Strengths and Limitations

Several strategies were used to ensure the credibility of this research as follows: triangulation of data sources (participants from 3 community levels, educational institutions, CSCom-U, and district hospital maternity wards), methods (document, focus groups, and interviews with different actors) and researchers (ie, triangulation of several analytical perspectives) to ensure consistency of the data and analysis; double coding for qualitative analysis; double member checking by sending preliminary results to participants belonging to the 3 target groups to validate and rectify them if necessary; skeptical peer review by members of the research team who will question methods interpretations, and meanings throughout the process [24]; detailed, rich, and concrete description [25] of the study context to enable readers or research users to judge potential transferability to other contexts, as well as a detailed description of the methodological approach, including the research site and data collection methods; and sample diversification to promote a wider application.

The resulting recommendations will address multiple stakeholders’ perceptions and needs, promoting alignment across various actors.

Finally, this research was developed through a collaborative and participatory approach, fostering an equitable partnership between research teams in Mali and the Canadian Consortium. Local researchers and partners led the selection of priority research questions and study design, ensuring that the research project reflected community priorities. Field researchers collected data while minimizing cultural bias, through an ongoing collaborative approach to interpretation between Malian and Canadian research teams. This approach involves local community representatives in several stages of the research process, including validation of tools, recruitment of participants and investigators, and validation of results analysis. To maximize the impact and accessibility of the research, results will be disseminated through many strategies. By focusing on local expertise and perspectives, this approach will promote greater ownership of the results by stakeholders.

However, some limitations were encountered during the data collection process. First, the inclusion criteria (15-19 years old and single) made access to adolescents in rural areas difficult. Most girls are already married by this age, and single girls often move to the city to work as domestic servants. Moreover, adolescents are rarely found at home, choosing instead to go to meeting places or sports activities. This required interviewers to seek them out in schools or conduct interviews in the evening. On the other hand, the availability of service users also posed a problem, requiring late-evening or late-day appointments. Finally, the complexity of the SRHR subject made the questionnaire lengthy, leading to a few complaints, although everyone completed the survey.

### Conclusion

In summary, using a concurrent mixed methods study, this needs assessment will help provide essential knowledge on how to align the nursing and medical curriculum with the needs of different levels of stakeholders (community, education, and health care providers), increase the capacity of future and current health care providers to deliver appropriate SRH services, and ultimately address the needs of the Malian community, reduce inequities and promote social justice.

## Abbreviations

ASACO: Associations de santé communautaire
CLEFS: Communautés locales d’enseignement pour les femmes et les filles en santé
CSCom-U: university community health centers
FMOS: Faculty of Medicine and Odontostomatology
FP: family planning
GBV: gender-based violence
GE: gender equality
INFSS: Institut National de Formation en Science de la Santé
M/F: male/female
PHC: primary health care
QDA: Qualitative Data Analysis
SRHR: sexual and reproductive health and rights
STI: sexually transmitted infection

## Data Availability

The datasets generated and analyzed during this study are not publicly available due to ethical and privacy considerations but are available from the corresponding author on reasonable request, if approval is obtained from the relevant ethics committee and data sharing complies with institutional and legal guidelines.
